# Efficient and scalable Python implementation of ANCOM-BC and ANCOM-BC2 for omics differential abundance testing

**DOI:** 10.1093/bioinformatics/btag767

**Published:** 2026-10-13

**Authors:** Zijun Wu, Huang Lin, James T Morton, Qiyun Zhu

**Affiliations:** Biodesign Center for Fundamental and Applied Microbiomics, Arizona State University, Tempe, AZ 85281, United States; Department of Epidemiology and Biostatistics, School of Public Health, University of Maryland, College Park, MD 20742, United States; Gutz Technologies, Inc., Covina, CA 91723, United States; Biodesign Center for Fundamental and Applied Microbiomics, Arizona State University, Tempe, AZ 85281, United States; School of Life Sciences, Arizona State University, Tempe, AZ 85281, United States

## Abstract

**Summary:**

Analysis of Compositions of Microbiomes with Bias Correction (ANCOM-BC) and its successor ANCOM-BC2 are critical algorithms for differential abundance analysis. Designed for handling compositional data, they feature the ability to estimate the unknown sampling fractions and correct the bias induced by their differences. While the original R package implementing the two methods is robust for standard dataset sizes, the increasing magnitude of modern multiomics studies presents computational challenges, often resulting in a runtime bottleneck for high-dimensional data. Here, we present native Python implementations of both ANCOM-BC and ANCOM-BC2 in the scikit-bio package, significantly accelerating the workflow and enabling their application to large-scale datasets. By leveraging vectorization with NumPy and numerical optimization in SciPy, we enhanced the efficiency of both methods without compromising their statistical accuracy. The benchmarking results revealed a dramatic increase in performance in both simulated and real datasets. We demonstrated that our Python implementations generated test results matching the original R code, while achieving 380- and 147-fold faster runtimes and 97.4% and 96.2% lower memory consumption on the largest tested dataset, respectively. The Python implementations allow for the analysis of datasets that are significantly larger than previously possible, and enable applications to alternative omics data types and complex statistical models that vary greatly by the number of samples, features, and covariates.

**Availability and Implementation:**

https://github.com/scikit-bio/scikit-bio

## 1 Introduction

Differential abundance (DA) is an important problem for omics-wide association studies under different conditions due to the complexity of high-throughput data. Analysis of Compositions of Microbiomes with Bias Correction (ANCOM-BC) is a foundational and widely used method for DA analysis in microbiome research (Lin and Peddada 2020). A key strength of ANCOM-BC is its ability to explicitly estimate unknown sample-specific sampling fractions and to correct the bias introduced by their variation across samples. This provides a statistically grounded approach to addressing confounders from unobserved absolute abundances, a common challenge in microbiome studies, where measurements are typically limited to relative abundances. The successor of ANCOM-BC, ANCOM-BC2 (Lin and Peddada 2024), further refines the sampling fraction estimation and bias correction procedure, incorporating additional sensitivity analysis for pseudocount addition, and multiple testing correction strategies to improve statistical power. By addressing the source of bias across samples, the two methods (collectively referred to as ANCOM-BC methods hereafter) enable more accurate inference of absolute feature abundances from compositional (i.e. relative abundance) data (Quinn *et al.* 2018). Similar limitations regarding compositional constraints apply to a variety of omics research fields, such as single-cell transcriptomics (Büttner *et al.* 2021), metabolomics (Krutkin *et al.* 2025), and glycomics (Bennett *et al.* 2025). Therefore, ANCOM-BC methods provide improved control of false discovery rates and greater interpretability compared with approaches that rely solely on relative abundances, making it an essential tool for rigorous and reliable DA analysis across the omics spectrum.

As omics workflows continue to scale in data complexity and integrate with machine learning paradigms, the impact of DA methods increasingly depends on their computational efficiency. In practice, DA serves not merely as a final statistical test, but as a repeated procedure of sensitivity assessment, cross-validation (Pelto *et al.* 2025), and dynamic feature selection step in predictive modeling (Quinn and Erb 2020, Romano *et al.* 2025). Consequently, efficient and scalable implementations are essential due to the increased computational cost in large-scale studies, enabling reproducible and flexible pipelines. This need motivates the development of Python-native implementations that ensure both computational enhancement and statistical accuracy.

Although bioinformatics software has traditionally been developed in R often, there has been a growing shift toward Python-based tools in recent years. Notable examples include the PyDESeq2 package (Muzellec *et al.* 2023) for differential gene expression (which is fundamentally equivalent to DA) analysis and the Scanpy framework for single-cell analysis (Wolf *et al.* 2018). This transition is driven by several advantages of the Python language, such as the access to well-maintained and efficient scientific computing libraries including NumPy (Harris *et al.* 2020) and SciPy (Virtanen *et al.* 2020), the integration with machine learning and deep learning frameworks like scikit-learn (Pedregosa *et al.* 2012) and PyTorch (Paszke *et al.* 2019), and the ability to reach a broader academic and industrial user base. Despite these benefits, until now no software provides Python-native DA analysis with explicit sampling-fraction bias correction. scCODA (Büttner *et al.* 2021) offers Bayesian compositional DA testing for single-cell data but does not estimate or correct sample-specific sampling fractions. A common workaround is to rely on Python-to-R interfaces, for example in the QIIME 2 platform (Bolyen *et al.* 2019), whereby R software is invoked from a Python environment. However, this approach complicates maintenance and deployment, and introduces computational overhead due to repeated data conversion and transfer between environments.

To address these limitations and fully leverage the advantages of Python-based software, we introduce native Python implementations of the two DA methods, ANCOM-BC (Lin and Peddada, 2020) and ANCOM-BC2 (Lin and Peddada 2024), both originally proposed and implemented in R. Using systematic benchmarking, we show that the Python implementations deliver significantly enhanced computational performance while preserving statistical accuracy.

## 2 Implementation

### 2.1 Algorithmic improvements

The key improvement in the Python implementations of the two ANCOM-BC methods focuses on efficiently solving the constrained optimization problem required to estimate the sampling bias inherent in compositional data. The algorithm iteratively solves for the bias term that minimizes the discrepancy in the log-ratios of abundances. The acceleration of our Python code is achieved through a comprehensive re-engineering of this bias estimation algorithm, transitioning from iterative processing to a fully vectorized framework. We used efficient linear algebra backends in NumPy on the entire input matrix simultaneously based on a detailed analysis of the underlying mathematical derivations and the original R code. In particular, we applied singular value decomposition just once to replace iterative maximum likelihood estimation, and reused its factors by extensive Einstein summation to solve for all features’ coefficients and their pseudoinverses simultaneously. The sampling bias correction step models the precorrection coefficients as a three-component Gaussian mixture: equally (75%)/less (12.5%)/more abundant (12.5%), consistent with ANCOM-BC’s assumption that true DA is sparse, and estimates a global bias term via expectation-maximization (EM). This procedure is still iterative, but the Python code vectorizes every E- and M-step across all features at once rather than looping feature-by-feature. While eliminating the computational cost of iterations, our code retains numerical accuracy identical to the original method. The rigorous mathematical translation is supported by SciPy’s efficient numerical optimizer for the variance estimation of parameters of a Gaussian mixture model at each M step, ensuring robust convergence without heavy computational redundancy. In addition, the codebase is distinguished by its maintainability. We included intensive discussions and mathematical derivations within the in-line comments, designed to facilitate future development as well as AI readability. The implementation is further improved by comprehensive unit tests that validate statistical correctness and handle edge cases, allowing the tool to remain scientifically reliable.

### 2.2 Software features

Two functions “ancombc” and “ancombc2” have been added to the Python package scikit-bio (Aton *et al.* 2026), with a simplified and versatile interface compared with the commands “ancombc” and “ancombc2” in the original R package ANCOMBC. Specifically, the Python functions consume a “table-like” object that supports various common formats, such as BIOM tables (McDonald *et al.* 2012), AnnData objects (Wolf *et al.* 2018), Pandas and Polars dataframes, and NumPy arrays. The statistical model is defined by providing the metadata table as a dataframe, and an R-style formula allowing for complex model definitions, such as the combination of categorical and numeric metadata columns and the inclusion of interaction terms. The behavior of the core ANCOM-BC algorithms is controlled by a small set of parameters, while the flexibility of data preprocessing is specified by the user. Both functions return a result object that supports a suite of *post-hoc* analyses matching the R workflow, including global tests, pairwise comparisons, Dunnett-type tests against a reference group, and order-restricted trend tests, computed on demand from the fitted model rather than at call time. A comprehensive tutorial with sample data and code is included in the documentation of “ancombc” to introduce the usage of this function together with data preprocessing and result postprocessing techniques to permit analysis consistent with the R package. In addition, a function “struc_zero” was added to test for structural zeros–features that are systematically absent from certain sample groups. This test was introduced along with the ANCOM-II method (Kaul *et al.* 2017) and has been incorporated into the R package ANCOMBC as part of the analytical workflow. In scikit-bio, we made it a standalone function as it is generally useful to the analysis of sparse omics data, with or without connecting to the ANCOM-BC tests. These two functions were placed along with an existing set of DA testing methods, including “ancom,” the predecessor of ANCOM-BC (Mandal *et al.* 2015), and “dirmult_ttest,” the Python reimplementation of an underlying method of another widely adopted DA package: ALDEx2 (Fernandes *et al.* 2014, Gloor *et al.* 2025). They share consistently styled interfaces to permit user experimentation with different methods. Scikit-bio also provides a comprehensive suite of compositional data manipulation tools, such as log-ratio transformation and multiplicative replacement, to facilitate power users to build customizable DA analysis workflows.

## 3 Benchmarking

### 3.1 Performance comparison on synthetic data

We evaluated the computational performance of our Python implementations in comparison with the original implementations in the R package ANCOMBC using synthetic datasets generated by the built-in simulator of the R package. Specifically, we employed a Poisson lognormal model that incorporates 10 continuous and 10 binary categorical covariates. This two-step simulation first sampled a latent Gaussian vector to establish taxon-to-taxon correlations and overdispersion, then drew observed counts from a Poisson distribution conditioned on the exponentiated latent values and a scaling factor. The variance–covariance matrix was estimated from the Quantitative Microbiome Project (QMP) dataset (Vandeputte *et al.* 2017) with 106 samples and 91 Operational Taxonomic Units (OTUs). The simulator finally outputs a 10 000 samples by 10 000 features count matrix along with 20 covariates metadata. The performance assessment was stratified into three distinct scenarios to isolate the impact of sample size, feature dimensionality, and covariate complexity. First, we assessed sample complexity by fixing the feature count to 1000 and covariates to 2, while incrementally increasing the number of samples. Second, we evaluated feature scalability by maintaining 1000 samples and 2 covariates while varying the number of features. We next examined the computational cost of model complexity by fixing both sample and features sizes to 1000, while increasing the number of covariates. The baseline configuration of 1000 samples, 1000 features, and 2 covariates was chosen over the full dataset to keep the runtime of the R code tractable. Finally, we ran the benchmarks on the full matrix consisting of 10 000 samples by 10 000 features with 10 covariates. For each scenario, we recorded both the runtime and peak memory usage, providing a direct comparison of resource utilization between the two methods under increasing computational loads.

Based on the benchmarking results across all tested dimensions, the Python implementations of ANCOM-BC methods achieved orders-of-magnitude acceleration in runtime alongside substantial reduction in peak memory allocation compared to the original R package (Fig. 1). Runtime speedup scaled strongly with the number of features and samples, reaching a peak of 302× for ANCOM-BC and 196× for ANCOM-BC2 at 10 000 features (Fig. 1A) and 103× speedup for ANCOM-BC and 50× for ANCOM-BC2 at 10 000 samples (Fig. 1B). This performance advantage extends to complex experimental designs, where the Python implementations achieved 30× to 46× speedup for ANCOM-BC and 12× to 15× speedup for ANCOM-BC2 in covariate-heavy settings (Fig. 1C). Complementing these runtime gains is an accompanying improvement in memory efficiency. The Python implementations decrease peak memory consumption by a 16- to 22-fold reduction for large datasets (6000–10 000 features or samples; Fig. 1D and E), and by >580-fold for ANCOM-BC and over 1400-fold for ANCOM-BC2 observed at the smallest dataset (10 features; Fig. 1D), where R’s peak usage is dominated by the fixed overhead of the interpreter and loaded packages. Across increasing numbers of covariates, memory reduction remained substantial for Python implementations, ranging from 44- to 56-fold for ANCOM-BC and 43- to 44-fold for ANCOM-BC2 (Fig. 1F). On the largest joint benchmark (10 000 samples by 10 000 features, 10 covariates), the Python implementation achieved a 380-fold runtime acceleration and a 97.4% reduction in peak memory for ANCOM-BC, and a 147-fold runtime acceleration and a 96.2% reduction in peak memory for ANCOM-BC2, relative to R. Importantly, across all tested settings in the simulated datasets, the Python implementations produced numerically identical estimates and DA results compared to the original R package, confirming that these performance gains were achieved without any loss of statistical accuracy. Collectively, the significant decreases in both runtime and memory usage in the Python implementation of ANCOM-BC methods validate its efficiency in analyzing massive omics datasets on standard hardware.

**Figure 1:**
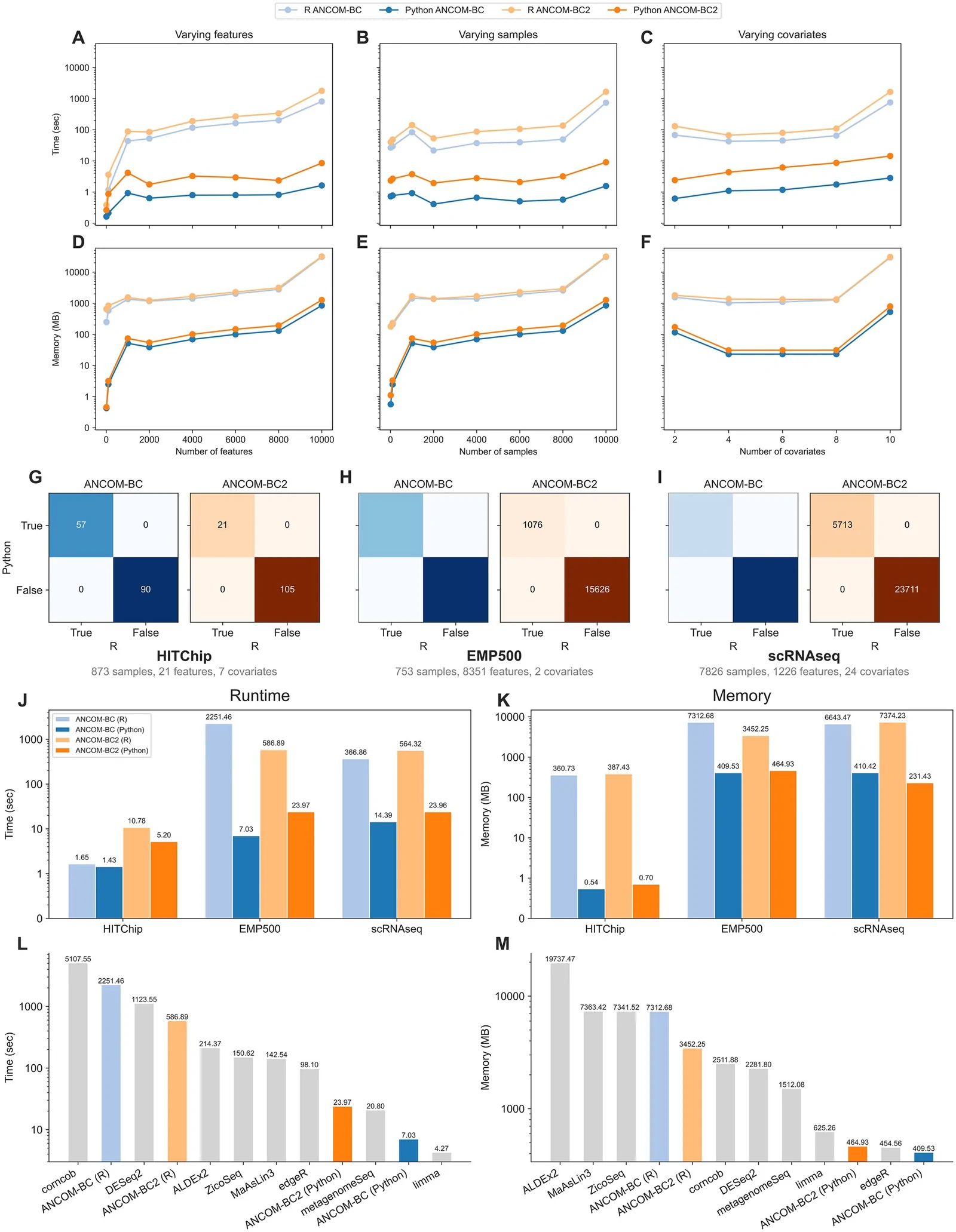
Runtime of R and Python implementations of ANCOM-BC and ANCOM-BC2 using synthetic datasets with varying numbers of features (A), samples (B), and covariates (C). Memory usage comparison between R and Python implementations on synthetic dataset with different number of features (D), samples (E), and covariates (F). Concordance between R and Python implementations and computational benchmarks across real datasets (G–I). Confusion matrices comparing DA calls made by the R and Python implementations on (G) the HITChip Atlas dataset, (H) the EMP500 dataset, and (I) the single-cell RNA-seq atherogenesis dataset. (J) Runtime and (K) memory usage of the R and Python implementations across the three datasets. (L) Runtime and (M) memory usage of the R and Python implementation of ANCOM-BC and ANCOM-BC2 ranked against other DA methods on the EMP500 dataset. Benchmark results were averaged over three repeated runs, using a single thread for each setting.

### 3.2 Applications on real-world omics datasets

To evaluate the Python implementations of ANCOM-BC methods across diverse biological contexts and data dimensions, we utilized three distinct datasets representing varying degrees of sparsity and complexity. The HITChip Atlas dataset (Lahti *et al.* 2014), demonstrated in the official ANCOM-BC tutorial, includes 873 samples and 21 features modeled against 7 covariates after preprocessing. To assess the performance in high-dimensional microbiome settings, we employed a filtered shotgun metagenomic dataset from the Earth Microbiome Project 500 (EMP500; Shaffer *et al.* 2022) comprising 753 samples and an expanded feature space of 8351 taxa, adjusted for 2 covariates. Finally, extending the framework beyond microbiomics, we included a single-cell RNA-seq dataset from an atherosclerosis study that contains 7826 cells, 1226 genes, and a complex experimental design involving 24 covariates (Park *et al.* 2025). The Python implementations returned concordant sets of differentially abundant features (Fig. 1G–I) while achieving greatly accelerated speed and reduced memory usage (Fig. 1J and K). To contextualize these gains relative to the broader DA analysis landscape, we compared the runtime (Fig. 1L) and peak memory usage (Fig. 1M) of the Python implementations of ANCOM-BC methods against a panel of commonly used DA methods [corncob (Martin *et al.* 2020), DESeq2 (Love *et al.* 2014), ALDEx2 (Fernandes *et al.* 2014, Gloor *et al.* 2025), ZicoSeq (Yang and Chen, 2022), MaAsLin3 (Nickols *et al.* 2026), edgeR (Robinson *et al.* 2010), metagenomeSeq (Paulson *et al.* 2013), and limma (Ritchie *et al.* 2015)] on the EMP500 dataset. These results demonstrate that the Python reimplementation not only resolves the runtime and memory bottlenecks but also positions ANCOM-BC and ANCOM-BC2 as among the most computationally efficient DA methods currently available without sacrificing the statistical rigor.

### 3.3 Benchmarking environment

All benchmarks were performed on a workstation equipped with an AMD Ryzen 9 9950X CPU, 128 GB of ECC DDR5 memory, and a PCIe 5.0 x4 SSD for storage. Software versions used were R 4.5.1, Python 3.13.5, and ANCOMBC 2.10.1. The specific version of the scikit-bio package used for comparison is noted in the Data Availability section, and all package versions (Python and R) are recorded in the archived benchmarking repository to support exact reproduction of the reported results.

## 4 Conclusion

The Python implementations of ANCOM-BC and ANCOM-BC2 in scikit-bio provide a robust and computationally efficient framework for DA analysis. By introducing this new feature, we aim to bridge a critical gap in the Python omics ecosystem and facilitate the integration of modern data science workflows into microbiome studies and beyond. With the enhanced performance of the ANCOM-BC methods, we unlock its potential as an explainable AI technique, particularly through the identification of discriminative balances (Quinn and Erb 2020). Coupling DA with machine learning models imposes a massive computational burden, high-efficiency implementations are therefore essential for accessing feature importance by sensitivity analysis and cross-validation. The future landscape of Python-based DA analysis is set to undergo major improvements driven by the needs for complex statistical modeling and high-performance computing. Sensitivity analysis and support for multigroup analysis are already available in the current implementation (Lin and Peddada 2024), and we are concurrently working on further advanced statistical modeling, including variance regularization. Upcoming developments will also prioritize sparse matrix operations for increasingly massive multiomics datasets, as well as data structure compatibility for an end-to-end bioinformatics workflow.

## Data Availability

The Python implementations of ANCOM-BC and ANCOM-BC2 have been incorporated into the scikit-bio package, available at: https://scikit.bio. The source code of scikit-bio is licensed under BSD-3 and hosted at the public GitHub repository: https://github.com/scikit-bio/scikit-bio. Version 0.7.4 of scikit-bio, which shipped the algorithms described in this work, has been permanently archived at Zenodo (DOI: 10.5281/zenodo.22880945). The benchmark datasets and scripts are available at: https://github.com/iiiime/ancom-bc_bm and have been permanently archived at Zenodo (DOI: 10.5281/zenodo.23272014).

## References

[btag767-B1] Aton M , McDonald D, Cañardo Alastuey J et al Scikit-bio: a fundamental Python library for biological omic data analysis. Nat Methods 2026;23:274–6. 10.1038/s41592-025-02981-z 41381898

[btag767-B2] Bennett AR , Lundstrøm J, Chatterjee S et al Compositional data analysis enables statistical rigor in comparative glycomics. Nat Commun 2025;16:795. 10.1038/s41467-025-56249-3 PMC11748655 39824855

[btag767-B3] Bolyen E , Rideout JR, Dillon MR et al Reproducible, interactive, scalable and extensible microbiome data science using QIIME 2. Nat Biotechnol 2019;37:852–7. 10.1038/s41587-019-0209-9 PMC7015180 31341288

[btag767-B4] Büttner M , Ostner J, Müller CL et al scCODA is a Bayesian model for compositional single-cell data analysis. Nat Commun 2021;12:6876. 10.1038/s41467-021-27150-6 PMC8616929 34824236

[btag767-B5] Fernandes AD , Reid JN, Macklaim JM et al Unifying the analysis of high-throughput sequencing datasets: characterizing RNA-seq, 16S rRNA gene sequencing and selective growth experiments by compositional data analysis. Microbiome 2014;2:15. 10.1186/2049-2618-2-15 PMC4030730 24910773

[btag767-B6] Gloor GB , Nixon MP, Silverman JD et al Explicit scale simulation for analysis of RNA-sequencing count data with ALDEx2. NAR Genom Bioinform 2025;7:lqaf108. 10.1093/nargab/lqaf108 PMC12362245 40837840

[btag767-B7] Harris CR , Millman KJ, van der Walt SJ et al Array programming with NumPy. Nature 2020;585:357–62. 10.1038/s41586-020-2649-2 PMC7759461 32939066

[btag767-B8] Kaul A , Mandal S, Davidov O et al Analysis of microbiome data in the presence of excess zeros. Front Microbiol 2017;8:2114. 10.3389/fmicb.2017.02114 PMC5682008 29163406

[btag767-B9] Krutkin DD , Thomas S, Zuffa S et al To impute or not to impute in untargeted metabolomics–that is the compositional question. J Am Soc Mass Spectrom 2025;36:742–59. 10.1021/jasms.4c00434 PMC11969646 40007142

[btag767-B10] Lahti L , Salojärvi J, Salonen A et al Tipping elements in the human intestinal ecosystem. Nat Commun 2014;5:4344. 10.1038/ncomms5344 PMC4102116 25003530

[btag767-B11] Lin H , Peddada SD. Analysis of compositions of microbiomes with bias correction. Nat Commun 2020;11:3514. 10.1038/s41467-020-17041-7 PMC7360769 32665548

[btag767-B12] Lin H , Peddada SD. Multigroup analysis of compositions of microbiomes with covariate adjustments and repeated measures. Nat Methods 2024;21:83–91. 10.1038/s41592-023-02092-7 PMC10776411 38158428

[btag767-B13] Love MI , Huber W, Anders S et al Moderated estimation of fold change and dispersion for RNA-seq data with DESeq2. Genome Biol 2014;15:550. 10.1186/s13059-014-0550-8 PMC4302049 25516281

[btag767-B14] Mandal S , Van Treuren W, White RA et al Analysis of composition of microbiomes: a novel method for studying microbial composition. Microb Ecol Health Dis 2015;26:27663. 10.3402/mehd.v26.27663 PMC4450248 26028277

[btag767-B15] Martin BD , Witten D, Willis AD et al Modeling microbial abundances and dysbiosis with beta-binomial regression. Ann Appl Stat 2020;14:94–115. 10.1214/19-aoas1283 PMC7514055 32983313

[btag767-B16] McDonald D , Clemente JC, Kuczynski J et al The Biological Observation Matrix (BIOM) format or: how I learned to stop worrying and love the ome-ome. Gigascience 2012;1:7. 10.1186/2047-217X-1-7 PMC3626512 23587224

[btag767-B17] Muzellec B , Teleńczuk M, Cabeli V et al PyDESeq2: a Python package for bulk RNA-seq differential expression analysis. Bioinformatics 2023;39:btad547. 10.1093/bioinformatics/btad547 PMC10502239 37669147

[btag767-B18] Nickols WA , Kuntz T, Shen J et al MaAsLin 3: refining and extending generalized multivariable linear models for meta-omic association discovery. Nat Methods 2026;23:554–64. 10.1038/s41592-025-02923-9 PMC12982127 41540124

[btag767-B19] Park C , Baek KI, Hung R-C et al Disturbed flow induces reprogramming of endothelial cells to immune-like and foam cells under hypercholesterolaemia during atherogenesis. Cardiovasc Res 2025;121:2679–99. 10.1093/cvr/cvaf233 PMC12860475 41288601

[btag767-B20] Paszke A , Gross S, Massa F et al PyTorch: an imperative style, high-performance deep learning library, arXiv, 2019, preprint: not peer reviewed.

[btag767-B21] Paulson JN , Stine OC, Bravo HC et al Differential abundance analysis for microbial marker-gene surveys. Nat Methods 2013;10:1200–2. 10.1038/nmeth.2658 PMC4010126 24076764

[btag767-B22] Pedregosa F , Varoquaux G, Gramfort A et al Scikit-learn: machine learning in Python, arXiv, 2012, preprint: not peer reviewed.

[btag767-B23] Pelto J , Auranen K, Kujala JV et al Elementary methods provide more replicable results in microbial differential abundance analysis. Brief Bioinform 2025;26. 10.1093/bib/bbaf130 PMC11937625 40135504

[btag767-B24] Quinn TP , Erb I. Interpretable log contrasts for the classification of health biomarkers: a new approach to balance selection. mSystems 2020;5:e00230–19. 10.1128/mSystems.00230-19 PMC7141889 32265314

[btag767-B25] Quinn TP , Erb I, Richardson MF et al Understanding sequencing data as compositions: an outlook and review. Bioinformatics 2018;34:2870–8. 10.1093/bioinformatics/bty175 PMC6084572 29608657

[btag767-B26] Ritchie ME , Phipson B, Wu D et al Limma powers differential expression analyses for RNA-sequencing and microarray studies. Nucleic Acids Res 2015;43:e47. 10.1093/nar/gkv007 PMC4402510 25605792

[btag767-B27] Robinson MD , McCarthy DJ, Smyth GK et al edgeR: a bioconductor package for differential expression analysis of digital gene expression data. Bioinformatics 2010;26:139–40. 10.1093/bioinformatics/btp616 PMC2796818 19910308

[btag767-B28] Romano S , Wirbel J, Ansorge R et al Machine learning-based meta-analysis reveals gut microbiome alterations associated with Parkinson’s disease. Nat Commun 2025;16:4227. 10.1038/s41467-025-56829-3 PMC12059030 40335465

[btag767-B29] Shaffer JP , Nothias L-F, Thompson LR et al; Earth Microbiome Project 500 (EMP500) Consortium. Standardized multi-omics of Earth’s microbiomes reveals microbial and metabolite diversity. Nat Microbiol 2022;7:2128–50. 10.1038/s41564-022-01266-x PMC9712116 36443458

[btag767-B30] Vandeputte D , Kathagen G, D’hoe K et al Quantitative microbiome profiling links gut community variation to microbial load. Nature 2017;551:507–11. 10.1038/nature24460 29143816

[btag767-B31] Virtanen P , Gommers R, Oliphant TE et al; SciPy 1.0 Contributors. SciPy 1.0: fundamental algorithms for scientific computing in Python. Nat Methods 2020;17:261–72. 10.1038/s41592-019-0686-2 PMC7056644 32015543

[btag767-B32] Wolf FA , Angerer P, Theis FJ et al SCANPY: large-scale single-cell gene expression data analysis. Genome Biol 2018;19:15. 10.1186/s13059-017-1382-0 PMC5802054 29409532

[btag767-B33] Yang L , Chen J. A comprehensive evaluation of microbial differential abundance analysis methods: current status and potential solutions. Microbiome 2022;10:130. 10.1186/s40168-022-01320-0 PMC9392415 35986393

